# The design and implementation of a novel music-based curriculum for dementia care professionals: The experience of SOUND in Italy, Portugal and Romania

**DOI:** 10.1186/s12909-024-05651-4

**Published:** 2024-06-17

**Authors:** Sabrina Quattrini, Alessandra Merizzi, Ioana Caciula, Lena Napradean, Maria João Azevedo, Sandra Costa, Sara Santini

**Affiliations:** 1 Centre for Socio-Economic Research On Aging, IRCCS INRCA-National Institute of Health and Science On Aging, Ancona, Italy; 2 Centru de Resurse Si Formare Profesionala, Asociatia Habilitas, Bucharest, Romania; 3 Scoala de Pian By Lena Napradean, Bucharest, Romania; 4 Associação Sons Do Estaminé, Trofa, Portugal

**Keywords:** Co-design, Dementia care professionals, e-learning platform, Evaluation, Music-based curriculum, Training

## Abstract

**Background:**

The positive effects of active and passive music activities on older people with dementia are well and largely documented by the literature. Nevertheless, the use of music as a non-pharmacological intervention is not so common both in private and public older people care facilities because in-home staff have no competencies for delivering such activities. Conversely, the realization and implementation of a co-designed music-based curriculum for dementia care professionals may help the diffusion of music in the older people care facilities. This study was aimed at evaluating the learning outcomes of the SOUND training, based on an original co-designed music-based curriculum for dementia care professionals and implemented in Italy, Portugal and Romania.

**Methods:**

The SOUND training study was developed through three phases: a) the co-design of the music-based curriculum for dementia care professionals, involving 55 people in the three participating countries; b) the teaching of the training curriculum to 63 dementia care professionals (29 in Italy, 17 in Portugal and 17 in Romania), delivered both in person and via a Moodle platform named Virtual Music Circle; c) the learning outcomes assessment, carried out by means of 13 self-evaluation tests, and a practical test, and the trainees’ course evaluation by a questionnaire.

**Results:**

Most of the trainees reached the highest score in the evaluation of the theoretical competencies in the three study countries. Conversely, some practical competencies in the facilitation of music activities need to be fine-tuned. The SOUND training course was evaluated very positively in the overall structure, theoretical contents, and practical workshops by the trainees. Nevertheless, they preferred the face-to-face compared to the distance learning methodology in the three countries.

**Conclusions:**

The SOUND training curriculum was effective in teaching music techniques and neurocognitive knowledge to dementia care professionals. Nevertheless, future courses should be differentiated for dementia care professionals with or without previous music knowledge and competencies. Moreover, the course is fully sustainable, because it does not require additional costs given that the curriculum is fully accessible online and it is also replicable because it trains professionals who can continue to apply the method in their working routine.

**Supplementary Information:**

The online version contains supplementary material available at 10.1186/s12909-024-05651-4.

## Background

According to the World Health Organization, there are currently over 55 million people living with dementia worldwide, and nearly 10 million new cases each year [1, 2]. These numbers are expected to more than triple by 2050 due to population growth and ageing [3], leading to considerable social and economic implications in terms of health and social care, informal care costs and public health systems sustainability [2].

To date, there are few pharmacological treatments for dementia and those available have a limited capacity to treat many of the symptoms of the disease and are often accompanied by side effects [4]. On the other hand, non-pharmacological treatments can delay functional decline and reduce the severity of behavioural and psychological symptoms of dementia [5], especially when combined with music-based interventions [6].

Passive and active music activities can have positive effects on older people with dementia’s behavior and mood [7], particularly agitation [8], anxiety [9] and depression [10]. Moreover, music can stimulate certain cognitive functions such as attention, language, and memory [11], particularly autobiographical memory, because music engages certain frontal brain areas that may be relatively preserved in most common dementias [12, 13].

Indeed, some skills such as learning new songs or detecting wrong notes in familiar songs are often spared in dementia [14]. Music is also a physical stimulus and engaging in physical exercise with music has been known to delay the onset of dementia [15–18]. Moreover, non-verbal behavior and sound-music performances used in music therapy enable participants to express their emotions and feelings, connect with others and improve communication and emotional status, with a positive adaptation to their social environment [6].

Despite the well-known effectiveness of music in the treatment of patients with dementia, and their advantages in terms of safety and costs compared to other non-pharmacological interventions [6, 19], the use of music-based activities is rarely or not at all included in the healthcare professional standard educational curriculum. Therefore, dementia care professionals are not skilled to lead music activities with patients in the working daily routine, unless they have followed music therapy studies in addition to the healthcare ones. As a consequence, the existing music-based interventions targeted to older people with dementia (OPDs) mostly require the involvement of music therapists as external experts [8, 11, 20].

The latter professionals are frequently paid “out of pocket” by the older guests unless music activities are delivered by volunteering organizations or funded through benefactors’ donations [19, 21], due to the limited resources allocated to music activities by public and private care providers [22].

To the best of our knowledge, there are only a few courses designed for non-musicians nor music therapists care professionals on the use of music with OPDs. One of them is reported by Stuart-Röhm et al. [23] describing the co-design of a “person-centered caregiver singing” (PCCS) protocol in which therapists shared music therapy-informed skills with caregivers for the use of music in daily care. Likewise, Ridder et al. [24] developed and tested a training manual to be used by music therapists to train DCPs in non-verbal communication with persons with late-stage dementia in residential care home contexts.

A recent Systematic Literature Review (Stuart-Röhm, Backer and Clark, 2023) [25] of studies reporting one-on-one relationship music trainings for formal dementia caregivers, highlights a great heterogeneity in this type of educational offer. Trainers included nurses or music therapists; trainees ranged between 5 and 26; every training was carried out in a different country; the trainings had different lengths ranging from a minimum of one hour to a maximum of five months; the music activities consisted mainly in singing favorite songs and making body movements; the style of the trainings included interactive lectures or videos. The reviewers observed and analyzed the effects of the training only on OPD treated by dementia care professional who had attended the training. The studies demonstrated that the use of music decreased agitation and disruptiveness, improved expression in emotions, and increased well-being of OPD. Nevertheless, neither the impact of the training on dementia professionals nor the learning outcomes were evaluated.

Less recently, a web-based music training for direct caregivers in long-term care communities, was combined with the implementation of the Music & Memory intervention [26]. The training was completed by 25 care professionals in four months. The training outcomes were monitored through a pre-post study.

McDermott et al. [27] underlined, among other issues, the importance of supporting the transition from music therapy interventions provided by music therapists to music therapy interventions provided by care professionals led or supervised by music therapists, the value of music therapy skill-sharing in training care home staff, and the importance of cultivating cross-professional dialogues to support organizational changes.

Another similar experience is that of the Circleactivities (CA),^1^ developed by Albert Hera and tested with eight OPDs for the first time in Trento (Italy) in 2018. The intervention, led by properly trained in–home staff (one nurse and one educator), showed positive effects on the coordination, mood, and well-being of older people with mild to moderate dementia [28]. Currently, the day-care center is applying music activities as part of the daily cognitive, sensorial, and social stimulation of the patients.

These good practices shed light on the appropriateness of providing dementia care professionals (not necessarily musicians) with competences for implementing music-based activities for improving and further humanizing the care provided to OPDs and for guaranteeing the diffusion and the long-term sustainability of non-pharmacological music interventions in older people care facilities.

The SOUND project, funded by the Erasmus + program, wants to cover this gap in the education of DCPs by: a) developing a curriculum on active and passive music-based activities for DCPs; b) delivering the training; c) testing an original non-pharmacological music-based intervention with OPDs; d) developing a “music for dementia” awareness campaign.

This paper describes how the SOUND training curriculum was co-designed and implemented in Italy, Portugal and Romania, and the system for evaluating the knowledge and the competences acquired by trainees, with the aim of inspiring further educational trainings in the use of music applied to the dementia care.

## Methods

**Fig. 1:**
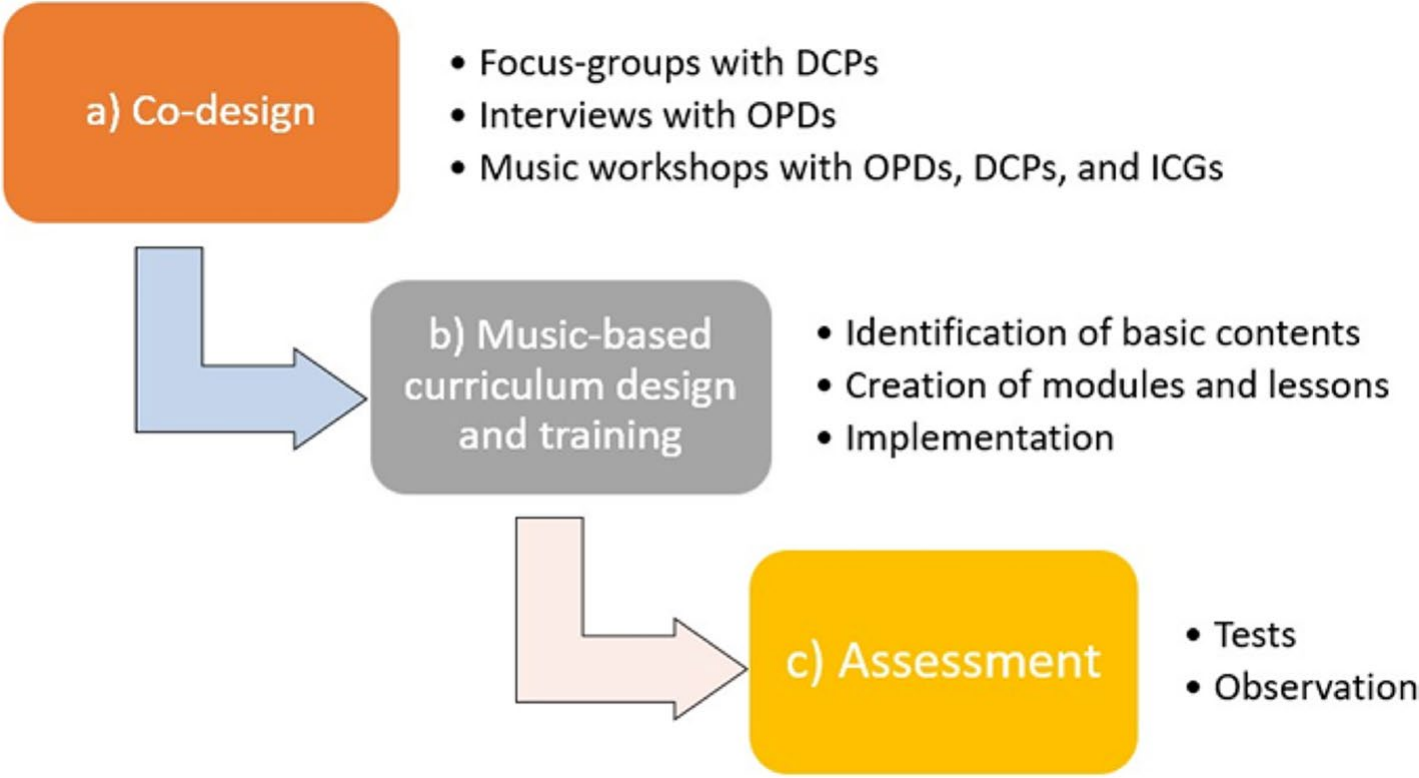
Training study design

The study developed through three phases: a) the co-design of the music-based curriculum for DCPs; b) the curriculum design and the delivery of the training; c) the assessment of the competences acquired by trainees (Fig. 1).

Every phase adopted a specific method, that is described below.

### The curriculum co-design

The curriculum was co-constructed with OPDs, DCPs and informal caregivers (ICGs) of OPDs, with the aim of identifying basic knowledge and competences to translate into the curriculum contents.

DCPs were included in the co-design process if they were working with dementia and were interested and motivated to take part in the study. ICGs were selected if they were the primary caregivers of OPDs for over a year, providing medium or intensive care (i.e.14–20 h of care per week). OPDs were included if they received a formal diagnosis of dementia and had a minimum Mini Mental State Examination (MMSE) score of 15; were able to express themselves verbally; did not present hearing and sight impairment nor motor problems that could not be helped through apposite aids and could prevent them from participating to the activities.

The co-design was based on the Experience-Based Co-Design (EBCD) method, a multi-stage process involving patients, caregivers and health care professionals in identifying how healthcare services can deliver enhanced experiences to improve care [29]. It embedded a practical and a research part.

The practical part encompassed two music workshops in each SOUND pilot country: the first with DCPs and OPDs and the second with DCPs, OPDs and ICGs.

A list of workshop activities was prepared by the national staff through the identification of main objectives for each group of participants to the co-design sessions, which are detailed in Annex 1 (Additional file 1). The main objectives were agreed by the consortium and then every national team adapted and personalized the activities based on their own resources, talents, and competencies. For example, in Romania, where the partner organization teaches mainly piano, the activities were thought to include piano live music for stimulating older people’s remembrances. Differently, in Portugal, where the partner organization teaches traditional folk music, the latter was adopted for the co-design sessions.

The research part was based on a pre-post study design according to which participants’ perspective was captured before and after the music workshops through different qualitative tools, namely: focus-groups with DCPs and ICGs, and individual interviews (in Italy) or focus-groups (in Portugal and Romania) with OPDs.

The topic-guides of the interviews and focus-groups are reported in Annex 2 (Additional file 2).

The interviews and focus-groups with the OPDs were led by an expert gerontologist or by a professional in the ageing field, with the support of care professionals such as psychologists and/or educators who knew the patients and worked in the Alzheimer or dementia care center.

In Italy and in Portugal, due to the cognitive condition of the OPDs, it was preferred to administer face-to-face individual interviews to avoid distractions and confusion as much as possible.

Interviews and focus groups were audio-recorded, then the content was transcribed verbatim by researchers at a national level in their country languages, and finally the main results were extrapolated as a list of tips for creating the training curriculum.

Every workshop comprised a “live” researchers’ observation of the participants’ reactions and behavior, and an “ex-post” observation made by researchers, facilitator and external observers from the video recordings of the sessions. The live observation encompassed the participation of at least two internal and three external observers.

The internal observers were asked to remember verbal and non-verbal reactions of participants to the stimuli given by the facilitator leading the activities for writing them down at the end of the session. The external observers had to observe the participants from outside the circle and note down observed face expressions, emotions, behaviors, and verbal expressions, by remaining “invisible”, i.e., not disturbing the interactions in any way.

A total of 55 people was involved in the co-design in the three participating countries (21 in Italy, 15 in Portugal and 19 in Romania), out of which 18 DCPs, 22 OPDs and 15 ICGs (Table 1). DCPs were all females (8 in Italy, 5 in Portugal and 5 in Romania). They were psychologists (8), healthcare staff i.e. educators and nursing assistants (5), and social workers (2). Half of them had between 10 and 20 years of experience in dementia. Only two professionals were also musicians.

**Table 1:** Participants in the SOUND co-design workshops per country (N/%)

	Italy (*N* = 21)	Portugal (*N* = 15)	Romania (*N* = 19)	Total (*N* = 55)
*Dementia care professionals*	*8 (44%)*	*5 (28%)*	*5 (28%)*	*18 (100%)*
Gender:				
Females	8 (100%)	5 (100%)	5 (100%)	18 (100%)
Males	0	0	0	0
Profession:				
Care staff member	2 (25%)	2 (40%)	0	4 (22.2%)
Professional educator	0	1 (20%)	0	1 (5.6%)
Geriatrician/Gerontologist	2 (25%)	0	1 (20%)	3 (16,7%)
Psychologist	4 (50%)	1 (20%)	3 (60%)	8 (44.4%)
Social worker	0	1 (20%)	1 (20%)	2 (11.1%)
Years of experience:				
< 10	1 (12.5%)	0	1 (20%)	-
10–20	4 (50%)	3 (60%)	2 (40%)	-
> 20	3 (37.5%)	2 (40%)	2 (40%)	-
Musician (Yes)	1 (20%)	1 (20%)	0	2 (11.1%)
*Older people with dementia*	8 (36%)	5 (23%)	9 (41%)	22 (100%)
Mean Age	79	77	75	-
Gender:				
Female	6 (75%)	3 (60%)	8 (89%)	17 (77%)
Male	2 (25%)	2 (40%)	1 (11%)	5 (23%)
Musician (yes)	0	1 (20%)	0	1 (4.6%)
*Informal caregivers*	5 (33%)	5 (33%)	5 (33%)	15 (100%)
Mean age	57	55	54	-
Gender:				
Female	3 (60%)	5 (100%)	4 (80%)	12 (80%)
Male	2 (40%)	0	1 (20%)	3 (20%)
Years of care:				
< 5	2 (40%)	2 (40%)	4 (80%)	8 (53%)
5–10	3 (60%)	0	1 (20%)	4 (27%)
> 10	0	3 (60%)	0	3 (20%)
Relationship with OPD:				
Daughter	2 (40%)	3 (60%)	4 (80%)	9 (60%)
Son	1 (20%)	0	1 (20%)	2 (13%)
Granddaughter	0	1 (20%)	0	1 (6.7%)
Spouse	2 (40%)	1 (20%)	0	3 (20%)
Working carer (yes)	1 (20%)	1 (20%)	3 (60%)	5 (33%)
Informal support (yes)	4 (80%)	4 (80%)	1 (20%)	5 (33%)
Formal support:				
Day-care centre	5 (100%)	3 (60%)	5 (100%)	13 (87%)
Home care	0	1 (20%)	0	1 (7%)

OPDs’ mean age was 77. Out of 22 OPDs, 17 were females and only one person had music skills.

Out of 15 ICGs, 12 were females (nine daughters and three wives). Six had been caring for 1–5 years; eight for 5–10 years and three for over 10 years. Only one third was working.

**Fig. 2:**
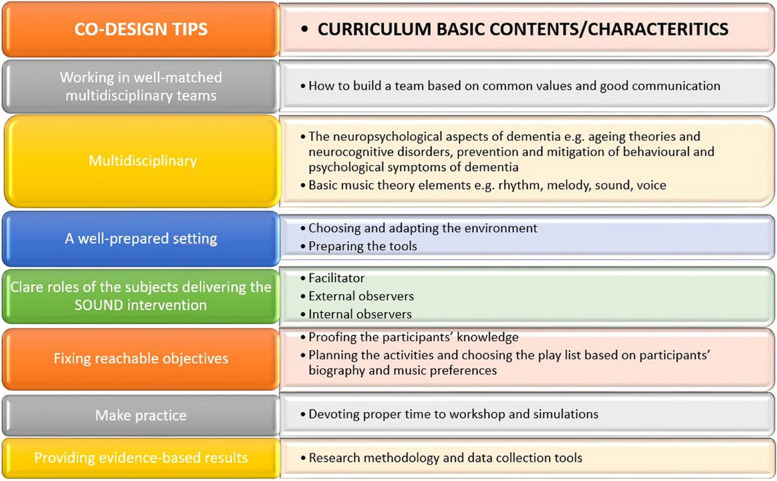
From the co-design to the curriculum basic contents/characteristics

The co-design sessions shed light on basic contents and characteristics of the SOUND curriculum and supported the formulation of relevant suggestions that shaped the SOUND curriculum framework, as depicted in the following Fig. 2.

### The SOUND curriculum

SOUND is a new and completely innovative method that does not aim to train new music therapists, but it is addressed to dementia care professionals to give them knowledge about how to use music in their work routine, as a means to improve the well-being of both themselves and OPDs. Thus, the researchers could just partly draw inspiration from the literature when planning the training. Moreover, similar trainings on music therapy for dementia professionals lasted from a minimum of one hour to a maximum of five months (Stuart-Röhm, Backer and Clark, 2023) not giving a clear indication on which was the more suitable and better length of a similar educational program.

So, the number of lessons and the amount of training hours were decided based on the time needed for teaching the core theoretical contents.

**Fig. 3:**
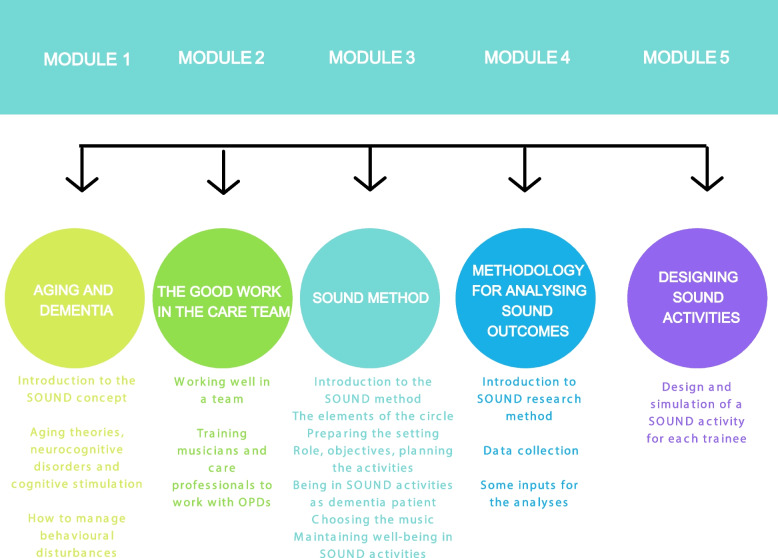
SOUND curriculum

Once the basic contents and priorities of the SOUND course were identified, the SOUND curriculum was built. It embedded six progressive modules (from 0 to 5) and 18 lessons for a total of 22 h of training (Fig. 3). Each Module was organized in a number of lessons corresponding to the topics to be covered and an assessment of how much time was necessary for either studying the documents provided (video, handout, PPT presentation) and attending to the online or F2F lessons and workshops.

The duration of the practical training especially, was established with the aim of giving the participants a reasonable time for recalling the theoretical contents learned during the online lessons, planning the activities, and carrying them on during the simulation of a SOUND session.

The curriculum was developed in English and translated in Italian, Portuguese and Romanian.

The training was carried out between June and July 2023 in a blended way: the 17 theoretical lessons (modules 0–4) were delivered online and the practical ones (module 5) face-to-face (F2F). The content was delivered via video-lessons, Power Point Presentations (PPT) and handouts via a Moodle platform called Virtual Music Circle (VMC) accessible at any time. Additionally, learners were required to complete a self-evaluation test (55 questions with either single or multiple choice) at the end of every lesson to assess their acquired knowledge and competencies, and a questionnaire to evaluate the training course, before they could obtain their attendance certificate.

The VMC was also conceived as a place to get and share resources in which DCPs can find examples of music tracks and music-based activities as well as can interact with each other through a Forum and a system for video calls.

### Analysis

As mentioned above, the study foresaw the assessment of the trainees’ knowledge and competences acquired throughout the course, and their evaluation of the training curriculum and delivery.

The theoretical learning was assessed by means of 13 self-evaluation tests, including 55 questions overall, undertook on-line at the end of the theoretical lessons (Annex 3-Additional file 3).

The self-evaluation tests were provided for all the asynchronous lessons except for the two lessons of module 0, relating to how to use the VMC, and one lesson of module 3 plus one of module 5 that were experiential and which learning was included in the other lessons and tests. Thus, the tests were 13 out of 17 lessons.

Trainees who attended 90% of the training hours and successfully completed all tests, each with a sufficient mark (8 +) and overall with a minimum mark of 80 (up to 131), obtained a certificate of attendance. The tests could be attempted multiple times until obtaining a pass mark.

Trainees’ competences (i.e. the ability for planning and leading music activities with OPDs) were evaluated by means of a practical test both over the course and at the end. During the F2F sessions (module 5) they were asked to invent and facilitate an activity in circle with a group of 5–6 other trainees simulating a session with OPDs and internal observers, while the trainers were evaluating their performances in the five domains as described in Table 2.

**Table 2:** Competences and skills: evaluation domains and meaning

Evaluation domains	Meaning
Setting and use of equipment	The ability to adequately prepare the setting that hosts the SOUND activities and use the equipment necessary to carry out the activity
Empathy and relationship	The empathy with which the facilitator proposed himself during the activity, and, at the same time, the ability to relate to each participant and to the whole group in the activity
Problem Solving	The capacity to resolve any alerts that arose during the circle, which includes the ability to realize that a problem had arisen, and to solve it while delivering the activity
Participation	The level of participation shown during the activity by the facilitator-trainee and the capability to face unexpected behaviors and difficulties simulated during the test
Creativity	The creativity to create and to manage, transform/remodel the ongoing activity based on the interpretation of eventual signals coming from users, including the capacity to re-propose the activity to the circle in a new and appropriate way, during the same SOUND session

Every domain was evaluated according to a 5-point scale (1 minimum, 3 sufficient and 5 maximum score). The sum of the scores gave a total score ranging from 5 to 25, where the threshold for the sufficiency was 15.

The trainees were observed by three trainers independently to minimize the risk of subjectivity in the evaluation of their performance. The scores from each trainer’s evaluation were averaged to obtain a total score for each student.

Concerning the evaluation of the training, as mentioned earlier, each trainee was required to provide a feedback at the end of the SOUND course through an evaluation form available on the VMC, for expressing their views on nine aspects of the training e.g., quality and usefulness of contents, the delivery modality, the e-learning platform, by using a 5-point scale (1 minimum-5 maximum) for each topic.

### Trainers and trainees’ recruitment and inclusion criteria

Trainees were recruited in the three participating countries between March and June 2023 using snowball sampling by disseminating a leaflet, containing the information about the course and how to apply, through various recruitment channels: a) a mailing list of professionals owned by national teams; b) a mailing list of the professional categories involved in the training (psychologist, educators, nurses, physiotherapists, doctors, etc.); c) Alzheimer day care centers, associations and nursing homes active at local, regional, and national level; d) social networks.

Trainees were included if they had a bachelor’s or a master’s degree in healthcare e.g. psychology, sociology, adult education, and were working as professional caregivers of people with dementia.

A simple descriptive analysis of socio-demographic data of trainees e.g. age, gender and type of care profession was made, and results were reported by frequencies and percentages. The learning outcomes score was calculated by every module and by country with the mere objective of underlining any possible differences at national level despite the homogeneity of the national samples (given by the inclusion criteria) and training contents. Given the very similar scores in the learning outcomes, ranging from 9.5 to 10 (Table 4), it did not make sense to calculate the significance.

## Results

### Trainees’ characteristics

A total of 63 DCPs attended a minimum of 90% of the total 22 h of training and passed the assessment phase: 29 in Italy, 17 in Portugal and 17 in Romania (Table 3).

**Table 3:** Trainees’ characteristics by country

Profession	Italy	Portugal	Romania	Total
Professional educator	12 (41.4%)	1 (6%)	1 (6%)	14 (22.2%)
Nurse assistant/Nurse	1 (3.4%)	14 (82.4%)	0 (0%)	15 (23.8%)
(Neuro)psychologist/psychotherapist	9 (31.1%)	2 (11.8%)	9 (52.9%)	20 (31.7%)
Other care workers	4 (13.8%)	0	7 (41.1%)	11 (17.5%)
Musician (not still working in the care sector)	3 (10.3%)	0	0	3 (4.8%)
Gender:				
Female	27 (93.1%)	15 (88.2%)	14 (82.3%)	56 (88.9%)
Male	2 (6.9%)	2 (11.8%)	3 (16.8%)	7 (11.1%)
Age:				
20–29	3 (10.3%)	5 (29.4%)	1 (6%)	9 (14.3%)
30–39	5 (17.3%)	2 (11.8%)	0 (0%)	7 (11.1%)
40–49	7 (24.1%)	7 (41.2%)	13 (76.4%)	27 (42.9%)
50–59	13 (44.9%)	2 (11.8%)	0 (0%)	15 (23.8%)
60–69	1 (3.4%)	1 (5.9%)	3 (17.6%)	5 (7.9%)
Living in:				
The same city as the course place	5 (17.2%)	6 (35.3%)	17 (100%)	28 (44.4%)
The same province as the course place	8 (27.6%)	9 (52.9%)	0 (0%)	17 (27.0%)
The same region as the course place	10 (34.5%)	1 (5.9%)	0 (0%)	11 (17.5%)
Another region as the course place	6 (20.7%)	1 (5.9%)	0 (0%)	7 (11.1%)
**Total**	**29**	**17**	**17**	**63**

In Italy, 39 people sent the application form to attend the training: 8 of them withdrew before or during the course and 2 did not complete the evaluation phase. In Romania, 20 people sent the application form to participate in the SOUND training course, of whom 17 attended and completed the course. In Portugal, 26 people applied to attend the training: 7 of them didn’t show-up for the first session (synchronous on-line), whilst 2 attended the first synchronous on-line session, but didn’t manage to continue with the F2F sessions.

Trainees belonged to four categories of professionals: a) professional educators, specialized in the education of adults with cognitive and/or physical impairment; b) (neuro)psychologists and psychotherapists; c) other care workers such as physiotherapist, podiatrist, junior doctor, manager of care facility, pharmacist, social care worker; d) musicians who were already or wanted to start working in older people care facilities. At a national level, trainees were mainly professional educators (41.4%) in Italy, three of which also musicians; nurse assistants or nurses (82.4%) in Portugal, and (neuro)psychologist/psychotherapists (52.9%) in Romania. Trainees were most females both at national and cross-national level (88.9%), aged mainly between 40 and 49 in Romania and Portugal (76.4% and 41.2%, respectively), and between 50 and 59 in Italy (44.9%).

Cross-nationally, 44.4% of trainees lived in the same city of the course, 27% in the same province, 17.5% in the same region and 11.1% came from different regions.

### Assessment of the learning outcomes

The self-evaluation of trainees’ theoretical learning resulted in a high total score at a country level (Table 4) with a low variability among the scores obtained for each lesson (ranging from 8 to 10). This depends on the fact that trainees could repeat the tests until they reached a passing mark. Self-evaluation tests were not available for two lessons of module 0, relating to how to use the VMC, one lesson of module 3 and one lesson in module 5.

**Table 4:** Trainees’ theoretical knowledge self-evaluation (mean score for each lesson)^a^

	Italy	Portugal	Romania
Module 1 – Lesson 1	9.7	9.6	9.7
Module 1 – Lesson 2	9.7	9.8	9.8
Module 1 – Lesson 3	9.7	9.5	10.0
Module 2 – Lesson 1	10.0	10.0	10.0
Module 2 – Lesson 2	9.7	9.8	9.9
Module 3 – Lessons 1, 2	9.5	9.7	9.8
Module 3 – Lesson 3	9.8	9.9	9.9
Module 3 – Lesson 4	9.8	9.9	9.6
Module 3 – Lesson 6	9.8	9.9	9.8
Module 3 – Lesson 7	9.9	10.0	10.0
Module 4 – Lesson 1	10.0	10.0	10.0
Module 4 – Lesson 2	10.0	10.0	10.0
Module 4 – Lesson 3	9.5	9.9	9.6
**Total score**	**128.3/130**	**127.0/130**	**128.9/130**

^a^The self-evaluation does not include the tests concerning lessons 1 and 2 of module 0 presenting how to access the VMC, and lesson 5 of module 3 consisting in practical exercises. Moreover, lessons 1 and 2 of module 3 had only one evaluation test

The evaluation of the ability to practice the SOUND method was based on the observation of a SOUND session facilitated by the trainees (Table 5). In Italy, 27 trainees were evaluated in their facilitation abilities: about three out of four reached the sufficiency in the total score (i.e. 15 +), 18.5% were assessed as “almost sufficient” (i.e. 14.5) and 7.4% were not sufficient. In Portugal and Romania trainees were evaluated with the majority achieving a total score of “good” (70.6%).

**Table 5:** Evaluation score of trainees’ competencies in the three countries (N, % of received scores)

	Italy	Portugal	Romania	Total
[17-25] Good	8 (29.7%)	12 (70.6%)	12 (70.6%)	32 (52.4%)
[15–16.9] Sufficient	12 (44.4%)	3 (17.6%)	3 (17.6%)	18 (29.5%)
[14.5] Almost sufficient	5 (18.5%)	0	0	5 (8.2%)
[11–14.4] Not sufficient	2 (7.4%)	2 (11.8%)	2 (11.8%)	6 (9.9%)
**Total Evaluated People**	**27**	**17**	**17**	**61**

When we look at the score on every assessed domain (Table 6), in Italy, the highest mean score was for “participation” (3.4) and “empathy and relationship” (3.2), and the lowest for “problem solving” and “creativity” (3.1). In Portugal, the highest average score was for “empathy and relationship” (4.2) and “participation” (4.2) and the lowest for “problem solving” (3.6). Romanian trainees achieved the highest score for “creativity” (4.0) and the lowest for “setting and use of equipment” (3.4).

**Table 6:** Evaluation of trainees’ competencies per country and per domain (mean scores, total scores, range)

	Italy	Portugal	Romania
Setting and use of equipment	3.0	3.8	3.4
Empathy and relationship	3.2	4.2	3.8
Problem Solving/Flexibility	3.1	3.6	3.5
Participation	3.4	4.2	3.8
Creativity	3.1	3.8	4.0
**Total Score**	**15.9**	**19.6**	**18.5**
Range of total scores	[11-21]	[11-25]	[12-25]

11 trainees did not receive a sufficient mark (i.e. 15) in the total score (seven in Italy; two in Romania; two in Portugal).

This part of the assessment did not affect the obtainability of the certificate of participation since it was meant to give the trainees a gradient on how skilled they might be as future facilitators and what are their strengths and weaknesses in the five dimensions at the end of the training. Those who have obtained the best scores will be taken into consideration for facilitating the SOUND activities during the intervention with OPDs.

### Trainees’ course evaluation

The SOUND training course was evaluated very positively by the Italian trainees, with mean scores higher than 4, thus more than sufficient, in each of the nine considered aspects (Table 7). Highest marks were for the effectiveness of the structuring of the topics and their logical sequence (4.5), and for the appropriateness of the navigation functions of the e-learning platform (4.5) (Table 7). Lowest marks were about: a) the use of an e-learning methodology appropriate to the topics covered (4.1), probably connected to the preference for interactive workshops (4.4); b) the usefulness of the gained knowledge for each participant (4.1); c) the consistency of the training activity as a whole, within the requirements of one’s professional role (4.1). Only three participants gave a non-sufficient mark (equal to 1 or 2) in 1 to 3 of the nine aspects of training they were asked to express their opinion, for a total of six not sufficient marks.

**Table 7:** Trainees’ course evaluation

	Italy	Portugal	Romania
1. Did the SOUND training meet your initial expectations?	4.3	4.8	4.8
2. To what extent were the stated objectives achieved?	4.4	4.8	4.9
3. How useful do you think the knowledge gained from participating in the training will be useful to you?	4.1	4.8	4.8
4. Was the training activity as a whole consistent with the requirements of your professional role?	4.1	4.8	4.8
5. Is the use of distance learning methodology appropriate to the topics covered?	4.1	4.1	4.5
6. Was the structuring of the topics and their logical sequence effective?	4.5	4.8	4.9
7. Were the navigation functions of the e-learning platform appropriate?	4.5	4.6	5.0
8. Were the forum and chat functions of the e-learning platform appropriately organized?	4.4	4.6	4.8
9. Were the interactive workshops effective in achieving the training course objectives?	4.4	4.8	5.0

The training course was evaluated very positively by the Romanian trainees, with mean scores higher than 4.5 in each of the 9 considered aspects, with highest marks in appropriateness of the forum and chat functions of the e-learning platform (5.0) and in the effectiveness of the interactive workshops for achieving the training course objectives (5.0). The lowest mark concerned the use of a distance learning methodology appropriate to the topics covered (4.5).

In Portugal, all training evaluation domains had a mean score of 4.8, except for the use of a remote learning methodology appropriate to the topics covered (4.1, being the lowest score), the adequacy of the navigation functions on the e-learning platform and the forum and chat functions on the e-learning platform (both with 4.6). Only 2 participants gave a score lower than 3 to the appropriateness of the use of distance learning for the topics covered.

## Discussion

SOUND is a multilingual and international curriculum on active and passive music making activities to use in dementia care settings targeted to DCPs with neither music therapy training nor music master’s degree. Thus, the SOUND course addressed the need for the autonomous use of music by professional care workers in the daily care routine without the direct intervention of music therapists, as suggested by the literature [23–25].

The DCPs who attended the SOUND training explored their own relationship with music and updated their knowledge on aging theories and neurocognitive disorders, particularly dementia, and on how to work in a multidisciplinary care team. They also learned how to bring music into the daily life of people they care for, how to plan and facilitate music activities, select music and combine it with activities that can stimulate the older person’s cognition and wellbeing, and observe the effects of the activities on older people as well as on themselves.

The self-evaluation questionnaires motivated trainees to go back to the lessons’ material for correctly answering the questions and obtaining a better score. The assessment of trainees’ conduction of one SOUND session showed that they gained enough skills related to music to use it in their work with OPDs, i.e. the ability to prepare the setting for delivering music activities; to invent music activities based on patients’ preferences, cognition and physical abilities (creativity); to facilitate/get involved in the activities (participation); the ability to understand the patients’ feelings and enter into a relationship with them through the music (empathy); the ability to use different type of music, communicate with internal operators and observers and modify the planned activities according to the patients’ current status for handling behavioural and psychological symptoms (problem solving-flexibility).

Note worthily, the self-evaluation scores are high and without variability cross-nationally because almost all trainees continued to repeat the test until they reached full marks. This outcome underlined the willingness of trainees to fully understand the SOUND concept.

Regarding the assessment of skills in the three countries, Italian trainees received lower marks than their Portuguese and Romanian counterparts. This aspect might be related to the fact that in Italy the evaluators were also the developers of the SOUND method for the entire consortium and had already experienced similar activities with a small group of seniors. Therefore, they probably had higher expectations on the performance of the trainees and were stricter in assessing their competences.

Concerning the course evaluation, the lower score assigned by Italian trainees to the course compared to Portugal and Romania, may depend on the composition of the group of trainees in Italy, characterized by a larger number of professional educators who also had prior knowledge of music (three trainees were musicians and three were also professional educators) and thus higher expectations of the course. This result calls for foreseeing some contents/lessons targeted to both DCPs and musicians and specific contents only for musicians for strengthening knowledge on ageing and dementia and only for DCPs for improving their music knowledge and competences.

Compared to the previous music trainings targeted to dementia care professionals [23–26], the SOUND training reached more care professionals, and it was carried out in three European countries (not only at national level). Moreover, the above-mentioned trainings foresaw face-to-face lectures or at distance lessons while SOUND included both the styles; the trainers were mainly nurses or music therapists while in SOUND the team of trainers included different professionals i.e. psychologists, musicians, sociologists, professional educators, and gerontologists. Furthermore, compared to similar music trainings, SOUND included not just singing and listening but also rhythmic exercises, body movement, narratives for stimulating memory and verbal fluency and adopted a kit of tools (e.g. balloons, little balls, Orff instruments, that may represent a powerful stimulus for OPD).

As for the strengths of the SOUND training, the main one is the bottom-up process that shaped its contents and structure, which gathered contributions from OPDs, DCPs and ICGs in the co-design workshop, and made it possible to mirror most of the trainees’ expectations.

Additionally, the SOUND training is fully replicable. In fact, the curriculum is available in four languages (English, Italian, Portuguese and Romanian) and it is fully free and accessible through the VMC platform. This makes it possible to train a great number of DCPs and musicians working from any location. Furthermore, the description of each activity led during the evaluation phase, was written, and summarized by trainee-facilitator (with all the participants in Portugal) in a predefined table, and it converged in a cross-national “Album of SOUND activities”, that will be at disposal of all the teams during the SOUND intervention. The “Album of activities” represents a rich source of inspiration for everyone who wants to replicate the method.

A further strength is the probable sustainability of the training, as suggested by the literature [19, 21, 22], linked to the likelihood for trained DCPs to apply the SOUND method in the care facilities where they work without additional personnel costs and with the only need to cover for low-cost materials e.g., balloons, foulards and sticks. The training is transferable because whoever will access the e-learning platform and complete the training autonomously through the VMC will be able to download a certification of frequency, after obtaining a minimum sufficient mark in each of the 12 tests (8 +) and overall (80 +), without taking part to the pilot training course. These new participants will not be able to implement the SOUND method because they lack the practical component of the training. Nevertheless, they may start to explore a new way to work with OPDs through the use of music and might want to attend further seminars for completing the missing experiential part of the training.

### Limitations

The SOUND training evaluation highlighted some limitations. The first is the lack of interviews to trainees for collecting their impressions on the training, that would have provided qualitative more informative data on their experiences with the training and the method. Moreover, the acquired competences should have been tested with OPD, because the simulation did not provide sufficient information on the real capabilities of facilitating a SOUND session with OPD. Since the sessions of the pilot intervention have been facilitated just by three out of the 63 dementia care professionals who attended the SOUND training, this small number does not allow to test the correlation between knowledge and competences acquired during the training and the real capability of trainees of properly applying the method and influencing the OPD’s well-being.

Another limitation of the study is the missed opportunity for assessing the retained knowledge at follow-up to verify the level of preservation of the learning. However, some trained dementia care professionals were selected to participate in the SOUND pilot study and had the chance to refresh their newly acquired knowledge by putting it into practice shortly after the end of the training. The SOUND training, like many other trainings, was designed for implementing the SOUND methodology soon after the teaching was completed. Thus, a follow-up evaluation of the acquired skills may be a valuable addition for those who will use the SOUND method in an undefined future.

In Italy, some trainees found the training not fully consistent with their profession. This might be connected to the novelty of the SOUND method and the absence of a benchmark for measuring the possibility to implement the acquired knowledge in one’s own profession. In Portugal and in Italy, trainees, but also trainers, considered that the time allocated for the synchronous and F2F sessions wasn’t enough for some topics, since active methods like simulations were often used to illustrate the practical elements of the training. In fact, such activities were considered a strong point of the training by participants. Further edition of the SOUND training and other music-based training for DCPs should foresee more practical activities e.g. workshops and less theoretical lessons.

## Conclusions

The study shed light on the fact that training DCPs on the use of music in daily care facilities may provide the opportunity to implement a non-pharmacological method for working with OPDs that is beneficial and sustainable thus cost-effective in terms of public health expenditure, and functional for improving the quality of the care provided to older people with dementia. Further studies are needed that show the potentialities disclosed by the use of music in residential and semi-residential care settings. The impact of the SOUND training on OPD’s well-being, mood, behavior, and cognition has already been tested through a quasi-experimental mixed-method research protocol [30] and the results (that will be reported in another paper) will hopefully contribute to increase the knowledge on the effects of music in the dementia care and shed light on the need of training.

### Supplementary Information

Additional file 1.

Additional file 2.

Additional file 3.

## Data Availability

The datasets generated and/or analysed during the current study are available from the corresponding author on reasonable request.

## Abbreviations

CA: Circleactivities
DCP: Dementia care professionals
EBCD: Experience-Based Co-Design
F2F: Face to face
ICG: Informal caregiver
MMSE: Mini Mental State Examination
OPD: Older people with dementia
PCCS: Person-centered caregiver singing
VMC: Virtual Music Circle

## Footnotes

1 Circleactivities 2018 by Alberto Quarello is licensed under Attribution-NoDerivatives 4.0 International.
