# Supplementary material for: The design and implementation of a novel music-based curriculum for dementia care professionals: The experience of SOUND in Italy, Portugal and Romania
Source: BMC Med Educ. 2024 Jun 17;24:668. doi: 10.1186/s12909-024-05651-4 (PMC11184888; doi:10.1186/s12909-024-05651-4)
Supplement: Supplementary file 1 — Additional file 1. [file 12909_2024_5651_MOESM1_ESM.docx]

**Annex 1**

**SOUND co-design workshops**

## **WORKSHOP IN ITALY**

- 1. **Workshop with DCPs and OPDs**

*Activity n. 1: “The names”, was aimed at introducing the facilitator and ice-breaking.* The facilitator welcomes (and proposes) to say your name starting from his and continuing counterclockwise. Then he invites them to stand up and sing or say their name in the way that everyone prefers and invites the circle to repeat the name of each person. The facilitator begins, and the others follow counterclockwise.

*Activity n. 2: “Sticks”* had the direct objective of stimulating the listening capability, attentiveness and coordination and the indirect objective of making the group. The facilitator, in an upright position like the rest of the circle, hands the sticks to the participants and illustrates the activity: facilitator will propose making sounds using the instruments and the participants will have to try to reproduce them.

*Activity n. 3: “Coloured foulards”* was aimed at stimulating coordination, body movement, sight, hearing and attention. The facilitator gives each participant a coloured foulard, trying to arouse general curiosity then, sitting down, then introduces the "game", asking the participants to hold the scarf with their right hand, when the facilitator raises her high up.

*Activity n. 4: “Story telling”*, was aimed at stimulating attention, memory and language fluency. The facilitator remains seated in the circle and introduces the activity, starting to tell that in the morning they saw something flying. Then the facilitator asks the participants questions to build a story together: each older person adds a detail, for example what is flying, and the participants agree that it is a kite, and then the colour of the kite, who makes it fly, etc

*Activity n. 5: “Body percussion”*, was aimed at investigating the body coordination, the memorization of the proposed movements, the attention required in carrying out the task, the inventive stimulation. The facilitator proposes a rhythm by clapping the hands and invites users to replay.

*Activity n. 6: “Listening to a song (“Grazie dei fior”) and association with colours”*. This activity aims to investigate how much memory and remembrance are part of the circle, using the visual and auditory aspect. The facilitator sits down to be part of the group listening to the popular song chosen for this activity: “Grazie dei fior” by Nilla Pizzi (the chosen song is in the key of Sidim): the circle listens, and the facilitator intervenes every now and again, and then the facilitator sings for inviting the members to do the same if they want. Some participants follow the music with their eyes closed, others do not. The co-facilitator follows the music by swinging the body left and right with marked movements: the facilitator asks "what colour did you come up with?" for soliciting the association between song lyrics and colours. The co-facilitator writes the colours on a poster placed on the ground.

*Activity n. 7: “Listening to a song”* (“Romagna mia”) was taught for visual, auditory, mnemonic stimulation and body coordination. The facilitator presents the new activity by saying that the participants will listen to a song that the facilitator listened to when they were little and went to dance with their parents, but does not remember the title, so they need the help of the group to remember it and tell their children too. After some minutes and some clues, the participants guess the title of the song: “Romagna mia”. The facilitator stands up, and invites the circle to stand up, in order to sing standing up and associating movements similar to the dance. The circle sings on the spot and follows the facilitator's movement, that is a wave movement, with the forearms forward swinging with the body, shifting the weight from the left foot to the right foot, alternating them for the duration of the verses of the song.

- 1. **The workshop with DCPs, OPDs and ICGs included five activities, described below.**

*Activity n. 1: “Sing your name”.* The facilitator starts introducing the activity, asks the group to stand up and grab each other’s hands, close their eyes, and listen to the silence and to leave all thoughts out of one’s mind. Then she proposes the group to sing or to say their names one after the other.

*Activity n. 2: “Listening to a popular song”.* The whole group is sitting down listening in silence, when the facilitator gets her bag and tries to look for something in the bag. She finds a song the group sings and follows the music. The facilitator encourages the group to move by saying “let’s move a bit” and by increasing her own movement.

*Activity n. 3: “Images of the city”* (Ancona). The facilitator is telling a story and the co-facilitator starts making soap bubbles generating a “wow” and “eh che bello!” by ITAOPD4, who then carries on suggesting the song “Le mille bolle blu”. So the facilitator welcomes the suggestion and begins to sing “Le mille bolle blu”, a successful song from Mina (a famous Italian singer), all the group sings with her. Then the facilitator goes back to her seat and carries on telling the story, the group keeps silent and listens. Then the facilitator stands up and goes around the circle asking each individual to choose an image specifying that everyone can look at them and get the one that they like most. At this point a game of imagination and remembrance starts, where every participant recalls personal or public events that occurred in the places represented in the photographs.

*Activity n. 4: “Coloured cloth”.* The facilitator shows a cloth coloured with different colours in triangular segments with a vertex in the centre of the cloth. They then get the cloth out and the facilitator asks each participant to grasp a flap. The facilitator remarks on the beauty of the cloth and begins to encourage an up and down movement accompanied by vocalisation “oooooh”. Everyone participates in having fun and asks everyone if they like the colour of the clothes each one has in front of him/her and what does it bring to their minds. The facilitator introduces the core exercise by listening to a beautiful song she has chosen for them “24.000 BACI”, which starts to play at 6:09, while ITAP9 starts moving the cloth producing various movements and the group follows, each one with preferred moves. Finally, the co-facilitator gets coloured balloons and throws them on the cloth. Everyone engages in the activity and, when some balloons get out of the cloth, several OPDs catch with one hand to bring them back into the center of the circle. The activity ends with a big applause from the whole circle.

*Activity n. 5: “Coloured balloons”.* The facilitator invites everyone to get a balloon and starts giving instructions, turned to the co-facilitator who is the first to do so, followed by all the others who take a balloon or are helped to do so by the facilitator. The latter explains the exercise: the facilitator calls a colour and the persons who have the balloon of that colour raise it. Then the facilitator explains to the group that they are going to listen to another song (“Finché la barca va”, a popular light song) and they will try to sing it.

## **Workshops in Portugal**

- 1. **Workshop with DCPs and OPDs**

*Activity n. 1: “The names and gestures”*, aimed to introduce the facilitator and break the ice. The facilitator welcomes and invites each person to say their name and invites the circle to repeat each person's name. Then invites them to say a name associated with a gesture, initially proposed by the facilitator and continuing clockwise. It resumes the activity but then in a counter clock wise direction.

*Activity n. 2: “Body movement imitation”* had the direct objective of stimulating memory, attention, coordination and the indirect objective of involving the group. The facilitator proposes movements that accompany a certain song and the participants must try to reproduce them. Then each participant presents a movement and everyone tries to imitate it.

*Activity n. 3: “Body Percussion”* aimed to stimulate attention, vision, hearing and coordination. The facilitator proposes the execution of rhythmic cells with the body (hands, feet, legs) and the group repeats. The facilitator gradually increases the degree of difficulty, checking the participants' response.

*Activity 4: “Sound Story”*, aimed to stimulate verbal fluency, association, memory and attention.The facilitator remains seated in the circle and introduces the activity, starting to tell how it has been to receive a special guest in her home. Then he asks the participants questions to build a story together.

*Activity n. 5: “Listen to a song”* (“Uma Casa Portuguesa” by Amália Rodrigues). This activity aims to promote auditory attention, memory, and explore verbal and emotional expression, through the use of a well-known fado in Portugal, which portrays Portuguese customs. The facilitator sits down to be part of the group listening to the chosen song chosen for this activity and the circle listens. Some participants follow along, singing parts of the song, others do not. The facilitator asks "what emotions did you feel?" and the co-facilitator writes the emotions described on a poster placed on the floor.

*Activity n. 6: “Dance and movement”*, aimed at visual, auditory, mnemonic stimulation and body coordination. The facilitator starts by presenting the music and proposes a movement to be followed by the group members. Then each element of the wheel proposes a movement and the rest imitate.

- 1. **Workshop with OPDs, DCPs and ICGs**

*Activity n. 1: “Imitation of body movement”* had the direct objective of stimulating memory, attention, coordination and the indirect objective of involving the group. The facilitator welcomes and proposes movements that accompany a certain song and participants must try to reproduce them. Then, each participant presents a movement and everyone tries to imitate it.

*Activity n. 2: “The names and sounds”*, aimed to introduce the facilitator and break the ice. The facilitator invites each person to say their name and invites the circle to repeat each person's name. Then invites them to sing your name by associating it with a gesture. Initially proposed by the facilitator and continuing clockwise through all the elements of the wheel. It resumes activity, but counterclockwise.

*Activity n. 3: “SPAM”* aims to stimulate memory, attention and verbal fluency. The facilitator proposes the execution of sets of syllables and the group members imitate. The facilitator gradually increases the degree of difficulty, proposing the intonation of the sets of syllables and associating them with facial expressions and gestures.

*Activity n. 4: “Music Listening.”* This activity aims to promote auditory attention, memory, and explore verbal and emotional expression. The facilitator plays a traditional Portuguese instrument "Cavaquinho", playing an original song, while the group listens attentively. At the end, the facilitator asks the circle members "what did they feel?", and there is a moment of sharing stories.

*Activity n. 5: “Singing a Song”*, aims to stimulate auditory attention, memory and verbal fluency. The facilitator and co-facilitator start playing the song "A Laurindinha", a traditional Portuguese song. The group starts singing the song, accompanying the facilitator and co-facilitator.

## **Workshop activities in Romania**

- 1. **Workshop with DCPs and OPDs**

*Activity n. 1: “Story telling”.* Designed as an ice-breaker, this activity sees the facilitator telling a story of a memory which involves children singing in a circle. She invites participants to sing with her just like the children were singing in the story. Then, together they start singing popular children’s tunes.

*Activity n.2: “The feeling of the song” (Elvis Presley - Can’t help falling in love)*. During this listening activity designed to trigger long-term memories and feelings, the facilitator invites participants to close their eyes and listen to a song. after the song finishes, participants are asked one by one what the song made them feel or think of.

*Activity n. 3: “Sing syllables”*. A short-term memory activity where the facilitator sings groups of syllables which the participants have to copy. The groups increase in complexity as the facilitator observes the opportunity.

*Activity n. 4: “Vowels and arm movements”.* A motricity and short-term memory activity where the facilitator starts a recording of a beat with harmony and listens to it for a bit while slowly moving their shoulder to the beat. She starts singing vowels, each one getting a different arm direction (e.g. a means arms up, e means arms outward, u means arms down, etc.). Participants have to copy the moves and sounds.

*Activity n.5: “Karaoke” (Ana zorile se varsa)*. Used to trigger reading abilities, in this activity the facilitator hands out lyrics to a song that all participants are familiar with. The song starts and everyone sings while reading the lyrics.

*Activity n. 6: “Vowels and arm movements WITH ADDED SHHHHH”*. A repetition of activity 4. with the aim of triggering the memory of just 10-15 minutes before. The facilitator starts a recording of a beat with harmony and listens to it for a bit while slowly moving their shoulder to the beat. She starts singing vowels, each one getting a different arm direction (e.g. a means arms up, e means arms outward, u means arms down, etc.). Participants have to copy the moves and sounds that they should be remembering from 2 activities ago.

*Activity n.7: “Dance - Francisco Canaro – Poema”*. A movement activity where the facilitator invites participants to stand up and while still in a circles, she does simple dance movements on the music, while participants copy the moves.

- 1. **Workshop with OPDs and ICGs**

*Activity n. 1: “Dance” (Stefan Banica - Imi acordati un dans).* A very forward starting activity meant to make everyone loosen up and open up - The facilitator invites participants to stand up and while still in a circle, she does simple dance movements on the music, while participants copy the moves.

*Activity n. 2: “Sing syllables” (Smooth jazz backing track)*. A lighter activity to warm up the voices, help with imitation skills, coordination and short-term memory - The facilitator sings groups of syllables which the participants have to copy. The groups increase in complexity as the facilitator observes the opportunity.

*Activity n. 3: “Listen to the kalimba & play the kalimba”.* To introduce novelty, the facilitator proposes the use of instruments in this workshop. The first one is a kalimba that she first plays as an example and then asks for the participants opinions on the sound that it makes, how it makes them feel, if it reminds them of anything from their past. She passes the instrument around the circle for everyone to try it out.

*Activity n. 4: “Explore percussion instruments*”. The facilitator hands out a different instrument to each participant and lets them freely play the instrument, to see how it sounds. She asks the participants to stop, then starts playing a beat which the participants have to follow. From here, different rhythms are being directed by the facilitator towards some participants at times or all of them at other times

*Activity n. 5: “Be the orchestra in a song”* *(Eugen Doga - Gramophone).*  Designed for coordination, listening skills and social interaction, in this activity Each participant has a percussion instrument and the facilitator directs the beat and rhythms to be played by the participants, in accordance with the music. Rhythms are repetitive, allowing the participants to feel at ease and enjoying listening to the song while also playing the instruments.

*Activity n. 6: “Dance” (Tina Turner - Proud Mary).* To close the circle of activities, the last one resembles the first one - the facilitator invites participants to stand up and while still in a circle, she does simple dance movements on the music, while participants copy the moves.
