# Supplementary material for: The design and implementation of a novel music-based curriculum for dementia care professionals: The experience of SOUND in Italy, Portugal and Romania
Source: BMC Med Educ. 2024 Jun 17;24:668. doi: 10.1186/s12909-024-05651-4 (PMC11184888; doi:10.1186/s12909-024-05651-4)
Supplement: Supplementary file 2 — Additional file 2. [file 12909_2024_5651_MOESM2_ESM.docx]

**Annex 2**

**Co-design data collection tools**

1. **The topic-guide of the focus-group with DCPs**

*Before the workshop:*

1. Which are the main difficulties you encounter as health care professionals in the field of dementia?
2. How do you feel at the end of a work shift?
3. What is missing in the care of the elderly with dementia?
4. Which are the greatest satisfactions you find in your work?

*After the workshop:*

1. How did you feel during the workshop?
2. How would you define the SOUND activities?
3. Do you think it is possible to apply the SOUND activities to the service/day-care centre where you are working and with patients you are caring for?
4. Which obstacles and barriers do you see to use the SOUND activities to your daily work with OPDs?
5. Which opportunities do you see in using the SOUND activities in your daily work with OPDs?
6. How can the SOUND activities help you in your job?
7. How should the SOUND activities be designed and thought about in order to contribute to your well-being?
8. How should the SOUND activities be designed and conceived in order to positively influence the team?
9. **The topic-guide of the focus-group with ICGs**

*Before the workshop:*

1. How do you feel at the end of the day?
2. Which are the main difficulties you encounter in caring for your family members with dementia?
3. Are there any positive aspects of taking care of your loved one? If so, which ones?
4. What is your relationship with music? May you describe it briefly??
5. What is your loved one’s relationship with the music? May you describe it briefly?
6. Do you use music as a strategy to handle your relative’s mood and behaviour in your daily care?

*After the workshop:*

1. How did you feel during the workshop?
2. How can the SOUND activities help you in your daily routine with your loved-one?
3. Which opportunities do you think there could be in using the SOUND activities with your relatives?
4. **The topic-guide of the interview (in Italy)^[[1]](#footnote-1)^ and focus-groups (in Portugal and Romania) with OPDs**

*Before the workshop:*

1. What is music for you?
2. What music was there in the most beautiful moments of your life?
3. Which music do you prefer?

*After the workshop:*

1. How did you feel during the workshop?

1. In Italy, the discussion with OPDs has been stimulated with:

   - Music, both used during the workshop and others: the mimic and behaviours of OPDs were observed while listening to the music.
   - Photographs were shown of people with different moods for grasping the OPDs’ feelings.

   [↑](#footnote-ref-1)
