# Supplementary material for: The design and implementation of a novel music-based curriculum for dementia care professionals: The experience of SOUND in Italy, Portugal and Romania
Source: BMC Med Educ. 2024 Jun 17;24:668. doi: 10.1186/s12909-024-05651-4 (PMC11184888; doi:10.1186/s12909-024-05651-4)
Supplement: Supplementary file 3 — Additional file 3. [file 12909_2024_5651_MOESM3_ESM.docx]

**SELF-EVALUATION TEST**

**Module 1: Aging and dementia**

**Lesson 1: Introduction to the SOUND concept**

**Please answer the following questions, by choosing one answer^[[1]](#footnote-1)^:**

**1.1.1 Treatments to combat dementia are** (one answer)**:**

1. Pharmacological treatments
2. Passive (listening) and active (creating sounds and rhythms) musical activities
3. Neurosurgery intervention
4. None of the above

**1.1.2 Among SOUND OBJECTIVES there are** (more than one answer):

1. Maintain cognitive function and improve behaviour, mood, and quality of life of informal caregiver of OPDs
2. Develop a methodology and a training curriculum for health workers of OPDs, based on active and passive music carried out in circle
3. Deliver an innovative intervention based on Circlesongs
4. Monitor the outcomes of the trial on the OPDs, informal caregivers and care professionals.

**1.1.3 THE SOUND INTERVENTION INVOLVES, IN EACH COUNTRY** (more than one answer):

1. Informal caregivers of OPDs
2. General Practitioners of OPDs
3. Migrant care workers of OPDs
4. Musicians

**1.1.4 Among the expected results of SOUND INTERVENTION there are** (one answer):

1. Reducing OPDs’ stress, anxiety and depression
2. Improving the wellbeing of all involved participants
3. Maintenance of residual cognitive abilities of OPDs
4. Improvement in the quality of the relationship between the informal caregiver and the OPD’s General Practitioner

**Module 1: Aging and dementia**

**Lesson 2: Aging theories, neurocognitive disorders and cognitive stimulation**

**Please answer the following questions, by choosing one answer:**

**1.2.1 What is ‘successful aging’?**

1. Working past the retirement age
2. Having hobbies in old age
3. Finding new social connections in old age
4. Normal, healthy aging and without disabilities, as much as possible

**1.2.2 The Socioemotional Selectivity Theory says that:**

1. As we age, we tend to become more involved in conflicts
2. As we age, we tend to avoid conflicts and experience positive emotions
3. As we age, we become more isolated
4. As we age, we tend to participate in less activities

**1.2.3 Neurocognitive disorders are:**

1. Part of the normal aging process
2. Occurring also in younger stages of life
3. Always accompanied by behavioural symptoms
4. Signs of stress

**1.2.4 Which of the following risk factors for dementia can be modified:**

1. Education
2. Parental family history of dementia
3. Aging
4. Genes

**1.2.5 Cognitive stimulation for people with dementia can be implemented through activities such as:**

1. Eating healthy food
2. Music activities
3. Walking
4. Taking medicines

**Module 1: Aging and dementia**

**Lesson 3: How to manage behavioural disturbances at home and in older people facilities**

**Please answer the following questions, by choosing one answer:**

**1.3.1 Which of the following is an example of Behavioural and Psychological Symptoms in dementia (BPSD)?**

1. Wandering and agitation
2. Memory loss
3. Loss of identity
4. Euphoria

**1.3.2 What is the percentage of people with dementia who will experience BPSD during the evolution of their disorder?**

1. 50%
2. 70%
3. 90%
4. 30%

**1.3.3 Sundowning means:**

1. Being hungry when sun is setting
2. The need of comfort felt by people with dementia
3. The confusion and agitation experienced by people with dementia when the sun is setting
4. Loneliness experienced by people with dementia during night

**1.3.4 Which of the following non-pharmacological interventions are among the most eligible for managing behavioural disturbances:**

1. Different activities
2. Offering sweet to the person with dementia
3. Music therapy
4. Occupational therapy

**1.3.5 According to research, for BPSD management, which of the following interventions are beneficial:**

1. Approaches that focus only on people with dementia, at home
2. Approaches regarding the environment only
3. Interventions focused on caregivers’ coping strategies
4. Talk therapy

**Module 2: The “good work” in a care team**

**Lesson 1: Working well in a team: input and tools for professionals**

**Please answer the following questions, by choosing one answer:**

**2.1.1 According to Harvard University, a well-managed collaboration has the following elements:**

a. Sufficiently executed

b. Led by the individual team participants

c. Time well spent

d. Occasional nourishment

**2.1.2 Questions that help us reflect on the concept of doing a good job include:**

a. What are my role models?

b. What are the principles that inspire the members of my work team?

c. Am I the professional my boss wants me to be?

d. When my boss speaks, does he/she address me most of the time?

**2.1.3 Lessons learnt from the Good Work Project include:**

a. The group leader must ensure that the views of the local/regional political class are taken into account.

b. The interest among team members must be mutual

c. Division of labour, setting of deadlines and mutual responsibility can be changed at any time

d. The team leader must give feedback even when it is not required

**2.1.4 Among the "warning signs" that indicate that a collaboration is not working are:**

a. Health problems of some team member

b. Excess of resources

c. Leadership problems

d. None of the above

**Module 2: The “good work” in a care team**

**Lesson 2: Training musicians and care professionals to work with OPDs**

**Please answer the following questions, by choosing one answer:**

**2.2.1 What methodology would you use to communicate with a person with severe cognitive language impairment?**

1. Cognitive stimulation
2. R.O.T (Real Orientation Therapy)
3. C.A.A. (Alternative Augmentative Communication)
4. Occupational therapy

**2.2.2 What does Burnout mean?**

1. Illness
2. Depression
3. Poor understanding
4. Exhaustion

**2.2.3 Crowdfunding is used to:**

1. Find new volunteers
2. Publicise training courses
3. Collect economic resources online
4. Collect economic resources in direct contact with the public

**2.2.4 With whom does the family have an initial interview in order to have their loved one admitted to the Day Care Centre?**

1. The coordinator/psychologist
2. The reference tutor
3. The team
4. The educator

**2.2.5 What does it mean that the Day Care Centre is highly "personality-intensive"?**

1. That it is fundamentally centred on the quality of human resources
2. Which has legal personality
3. Which works with disabled people
4. Which is part of the Third Sector

**Module 3: SOUND method**

**Lesson 1: Introduction to the SOUND method: circle concept and the Circleactivities**

**Lesson 2: The elements of the circle: facilitator, observers and beneficiaries**

**Questions on lessons 3.1 and 3.2**

**3.1-2.1 What are the basics of Circleactivities? (more than one correct answer):**

1. Voice and singing

2. The large group dimension

3. The function of the circle

4. The use of musical instruments

**3.1-2.2 Of the following, which are part of the facilitator's languages (one correct answer)?**

1. Verbal, non-verbal, ideomotor

2. Verbal and motor

3. Paralinguistic, chronological, kinaesthetic, proxemics

4. Non-verbal and figurative

**3.1-2.3 How many areas comprise spatial behaviour (one correct answer)?**

1. Two: Posture and movement

2. Three: Area delimitation, eye contact and physical contact

3. Three: Orientation, posture and distance keeping

4. Four: Contact, orientation, proxemics and posture

**3.1-2.4 What are the facilitator's circles (one correct answer)?**

1. The circle of the self

2. The circle of seeking

3. The activity circle

4. All of the above

**3.1-2.5 The figure of the facilitator is based on (one correct answer)**

1. on improvisation, creativity, strategy, methodology and observation

2. exclusively on methodological analysis

3. exclusively on improvisation

4. on improvisation and observation

**3.1-2.6 The research circle (one correct answer)**

1. is formed exclusively by the staff of researchers

2. involves collaboration between researchers and facilitator

3. gives the facilitator the objectives to be pursued with the activity

4. has the task of intervening during the activity to support the facilitator

**3.1-2.7 On which of the following macro-areas is the Big Five theory based (one correct answer)?**

1. Professional competences

2. Ability to recognise one's own emotions

3. Creativity

4. All of the above

**3.1-2.8 The facilitator (more than one correct answer)**

1. has responsibility for the entire activity

2. must seek constant training and lots of practice

3. must understand his/her own limitations

4. must memorise the strategies included in the project

**Module 3: SOUND method**

**Lesson 3: Preparing the setting for the Circle: the environment, the tools and the inclusion of the participants. Practical exercises.**

**3.3.1 Seats in the circle (more than one correct answer)**

1. Are prepared by the facilitator in advance

2. Are random, depending on the order in which users arrive in the room

3. They take into account interpersonal dynamics

4. Each person can choose where to sit

**3.3.2 The users to be involved are chosen on the basis of:** (one correct answer)

1. Cognitive status

2. Presence of emotional-behavioural symptoms

3. Sensory status

4. All of the above

**3.3.3 In order to correctly choose the persons to be included in the activity, it is important to (more than one correct answer)**

1. That the user has a personal knowledge of music

2. To study the person's biography

3. The user must declare to the facilitator that they are interested in the activity

4. Make a good observation of the activities carried out and the degree of socialisation

**3.3.4 The activities proposed by the facilitator** (one correct answer)

1. There are about ten in total

2. They follow a precise order to prevent the facilitator from getting confused

3. They must allow flexibility to change the order of the proposal

4. They energetically stimulate the physical part

**Module 3: SOUND method**

**Lesson 4: Roles, fixing the objectives and planning the activities for delivering SOUND sessions: practical instruments**

**3.4.1 Which of the following are characteristics a facilitator should have?** (more than one correct answer)

1. Professional skills and creativity

2. Accuracy

3. Decision-making skills

4. Ability to recognise one's own emotions

**3.4.2 What are the tasks of the internal observer?** (more than one correct answer)

1. To participate emotionally in the experience

2. Take care of the people sitting next to him/her

3. Interrupt the facilitator and propose another activity if he/she notices particular behaviour in the users

4. All of the above

**3.4.3 The activities of a SOUND session:** (one correct answer)

1. Aim at improving well-being

2. Aim at improving socialisation

3. They arise from the combination of all elements collected during user profiling

4. All of the above

**3.4.4 The exercises offered in a SOUND session must also take into account** (more than one correct answer):

1. The musical preferences of the people with dementia in the group

2. The time available for internal observers

3. The knowledge and training of the operators

4. None of the above

**Module 3 – The SOUND Method**

**LESSON 5: Being in a sound activity as a dementia patient: putting professionals in the OPDS' shoes (workshop)**

No evaluation questions are asked for lesson 5 because it is a workshop.

**Module 3: SOUND method**

**Lesson 6: How to choose the music for the SOUND sessions: cultural aspects at country level**

**3.6.1 What are the functions of music in a Sound session?** (one correct answer)

1. Stimulus for thought associations

2. Dance stimulus

3. Rhythm stimulus with instruments

4. All of the above

**3.6.2 Music in a Sound session can be used for:** (one correct answer)

1. Create a recreational moment for the group

2. Change the mood

3. Distract participants

4. None of the above

**3.6.3 How can the musical tastes of older persons with dementia be known/identified** (more than one correct answer)

1. Given the pathology it is not possible to investigate this aspect

2. By collecting the biographies of older people with dementia

3. It is not relevant to know the musical tastes of older people with dementia but it is relevant to know those of their informal caregivers

4. Observing older people with dementia during musical activities

**3.6.4 The use of music with people with cognitive impairments** (more than one correct answer)

1. It is a tool that can be used by everyone easily and without special care

2. It is effective because the musical capacity is among the last to be totally impaired

3. It is effective because the brain areas associated with musical memory suffer less damage than areas associated with other memories.

4. It is to be used because it makes the activity fun

**Module 3: SOUND method**

**Lesson 7: Maintaining well-being in Circle Activities: how to prevent and hold uncomfortable situations in the SOUND group**

**3.7.1 What are the types of frustrations experienced by people living with dementia (more than one correct answer):**

1. It is not possible to know because people with dementia cannot express themselves
2. They are subjective and relate to the limiting symptoms of dementia, such as loss of memory, of autonomy, of being able to express themselves
3. There are no frustrations in living with dementia
4. Sometimes there are also frustrations regarding the stigma attached to the diagnosis and how others change when interacting with the person with dementia

**3.7.2 In group situations, the feeling of discomfort may increase because (one correct answer)**

1. The group is fine and having fun
2. The person does not want to participate in the activity
3. The person wants to participate but is limited in doing so and does not receive the support to participate with his/her limitations
4. The activity is a bit boring for that person

**3.7.3 What is important to consider in order to avoid uncomfortable situations (more than one correct answer):**

1. That the facilitator chooses the activities he/she likes best
2. That all professionals involved know all participants well, from their tastes to their vulnerabilities
3. That you know how to observe and support individual participants while having fun with them
4. To immerse oneself in the activity

**3.7.4 The tools for intervening in the case of increasing discomfort of a group member are: (one correct answer)**

1. empathy, the ability to give support, flexibility, non-verbal communication
2. remain indifferent to the person, as he/she can manage on his/her own
3. encouraging him/her to continue the activity as if nothing happened
4. putting him/her at the center of the group's attention

**Module 4: Methodology for analysing the SOUND outcomes**

**Lesson 1: Introduction to the SOUND research method: concept, participants’ selection and professionals’ identification**

**4.1.1 What are the inclusion criteria for the participants living with dementia: (one correct answer)**

1. Everyone who is interested can participate in the study
2. People with a diagnosis of Alzheimer’s disease who like music
3. Have a good enough cognitive state (MCI-mild dementia), don’t have much sensorial difficulties, are able to express themselves, understand and do simple tasks, are over 65 and keen to be in a group
4. All of the above

**4.1.2 What information is important to collect and learn for each OPD participant: (one correct answer)**

1. Their biography and music preferences
2. Their interests and habits
3. About their dementia history, their fears, and behaviours
4. All the above and more

**4.1.3 What is the role of the internal observer? (one correct answer)**

1. To help the facilitator deliver the activities
2. To have fun for the good of the SOUND activity
3. To learn all the songs and sing them out loud
4. To actively participate, keep an eye on the OPDs around them, collaborate with the facilitator and report their observations at the end of the SOUND activity

**4.1.4 Why researchers are involved in SA (one correct answer)**

1. Because SOUND is based on a scientific approach
2. Because researchers want to have fun
3. Because research funding is available
4. Because researchers are curious about the SOUND activity

**Module 4 – Methodology for analysing the SOUND outcomes**

**Lesson 2:** **Data collection: how to gather feedback from observers, facilitator and participants**

**4.2.1 When are the SOUND data collected? (one correct answer)**

1. Pre and post intervention
2. Data are only collected by researchers
3. Pre intervention, during the SA and post intervention
4. Pre intervention, during every SOUND activity, immediately after each SOUND session, two weeks post intervention

**4.2.2 Who is going to collect the data? (one correct answer)**

1. Only researchers
2. The internal and external observers
3. All professionals involved
4. Researchers, internal and external observers

**Module 4 – Methodology for analysing the SOUND outcomes**

**Lesson 3: Method for data collection and some inputs for the analysis**

**4.3.1 Why is the thermometer of emotions important? (more than one correct answer)**

1. Because it has a nice picture of the thermometer
2. Because it is a good way to add observations not included in the AARS
3. Because the internal observer can report how situations of discomfort felt and were handled
4. Because it is useful to learn and improve the quality of SOUND activities

**4.3.2 What is relevant to consider when introducing SOUND to your organisation? (more than one correct answer)**

1. Organise a meeting to show the method and highlight the useful and appealing aspects
2. What concerts are in the area
3. Organise a focus group to discuss aspects around workload, work satisfaction, music experience and music interventions
4. Plan all SOUND activities

1. In red the right answer(s) [↑](#footnote-ref-1)
